# Antimicrobial Porous Surfaces Prepared by Breath Figures Approach

**DOI:** 10.3390/ma11081266

**Published:** 2018-07-24

**Authors:** Alexandra Muñoz-Bonilla, Rocío Cuervo-Rodríguez, Fátima López-Fabal, José L. Gómez-Garcés, Marta Fernández-García

**Affiliations:** 1 Instituto de Ciencia y Tecnología de Polímeros (ICTP-CSIC), C/Juan de la Cierva 3, 28006 Madrid, Spain; 2 Facultad de Ciencias Químicas, Universidad Complutense de Madrid, Avenida Complutense s/n, Ciudad Universitaria, 28040 Madrid, Spain; rociocr@quim.ucm.es; 3 Hospital Universitario de Móstoles, C/Río Júcar, s/n, Móstoles, 28935 Madrid, Spain; flopezf@salud.madrid.org (F.L.-F.); jlgarces@microb.net (J.L.G.-G.)

**Keywords:** antimicrobial coatings, porous surfaces, breath figures

## Abstract

Herein, efficient antimicrobial porous surfaces were prepared by breath figures approach from polymer solutions containing low content of block copolymers with high positive charge density. In brief, those block copolymers, which were used as additives, are composed of a polystyrene segment and a large antimicrobial block bearing flexible side chain with 1,3-thiazolium and 1,2,3-triazolium groups, PS_54_-*b*-PTTBM-M_44_, PS_54_-*b*-PTTBM-B_44_, having different alkyl groups, methyl or butyl, respectively. The antimicrobial block copolymers were blended with commercial polystyrene in very low proportions, from 3 to 9 wt %, and solubilized in THF. From these solutions, ordered porous films functionalized with antimicrobial cationic copolymers were fabricated, and the influence of alkylating agent and the amount of copolymer in the blend was investigated. Narrow pore size distribution was obtained for all the samples with pore diameters between 5 and 11 µm. The size of the pore decreased as the hydrophilicity of the system increased; thus, either as the content of copolymer was augmented in the blend or as the copolymers were quaternized with methyl iodide. The resulting porous polystyrene surfaces functionalized with low content of antimicrobial copolymers exhibited remarkable antibacterial efficiencies against Gram positive bacteria *Staphylococcus aureus*, and *Candida parapsilosis* fungi as microbial models.

## 1. Introduction

Healthcare-associated infections are a major problem nowadays, causing high morbidity and mortality rates and substantial increase in health care costs. These infections are mainly associated with surgery procedures and medical devices such as ventilators or catheters. Prescription of antibiotics is typically used as a prevention method and/or treatment to avoid such transmissions of nosocomial pathogens; however, antibiotic consumption is a primary cause of antibiotic resistance [1], and consequently, there is an urgent need for alternatives, as well as strategies to prevent healthcare-acquired infections [2]. Inhibition of bacterial growth on the surfaces of medical equipment and devices is necessary to prevent the transmission of diseases by contact, and one promising approach is the development of antimicrobial coatings. Many of these self-disinfecting coatings are based on impregnating the surfaces with antimicrobial agents including antibiotics, silver compounds, light active species, and antimicrobial polymers such as polycations [3,4,5,6,7]. Other strategies limit the bacterial colonization of surfaces by inducing micro and nano-roughness, which modifies the surface area of contact with the microorganisms [8,9,10]. Although in the past the effect of surface topography on bacterial inhibition has received less attention, it is gaining popularity nowadays [11,12]. Many of these works study antifouling effects [13,14] based on superhydrophobic surfaces with reduced surface contact [10,15]. Most of the antifouling surfaces are able to effectively reduce the initial bacterial attachment, but only within a relatively short period. If adhesion occurs, the bacteria rapidly proliferate, leading to the formation of the biofilm.

Alternatively, the introduction of roughness can produce the opposite effect, dramatically increasing the contact adhesion area. Thus, combinations of surface roughness with chemical biocidal functionalities can create more effective bactericidal properties than flat surfaces [16,17,18]. Nowadays, many techniques are available to create surfaces with finely controlled topography, including lithography approaches and colloidal templates [19,20]. Most of these techniques usually require multiple stages, expensive equipment and prefabricated masks. One of the most versatile and simple methodologies to create polymeric porous surfaces with controlled pore size, and thus tailored roughness, is the so-called breath figures approach [21,22,23,24]. This method prepares ordered porous films with water droplets as the template. Basically, polymer solution is cast onto a substrate under humid atmosphere. The solvent evaporation induces the condensation of water droplets, which self-organize into a hexagonal array and after solvent evaporation, honeycomb-patterned films are obtained. Although this technique can be used with a diversity of polymers and functionalities [25], it is limited to polymers soluble in non-polar organic solvents such as CS_2_ or chloroform, which are mostly hydrophobic polymers, polymers with polar end groups or some amphiphilic copolymers. An alternative for obtaining porous surfaces functionalized with highly hydrophilic polymers or polyelectrolytes is the use of polymer blends, consisting of incorporating low amount of hydrophilic polymers into a hydrophobic polymeric matrix such as polystyrene [26]. Due to the formation mechanism of the breath figures, the hydrophilic polymers tend to migrate towards the condensed water droplets, which imply their localization at the surface, on the wall of the pores [26,27,28]. In this context, cationic antimicrobial polymers based on quaternary ammonium groups have been incorporated into breath figures films by using blends [29]. However, only systems based on copolymers with low positive charge density have been prepared due to the difficulty to dissolve them in organic solvents [29,30]. In this work, we prepared porous films by a breath figures approach functionalized with high charge density by the incorporation of antimicrobial cationic polymers bearing two quaternary ammonium groups per monomeric unit. In fact, these structures are based on methacrylic monomers with 1,3-thiazolium and 1,2,3-triazolium side-chain groups, and have demonstrated a broad spectrum of antimicrobial activity in solution [31,32], and also when immobilized onto a surface [16,33]. It is well known that surface positive charge density is an important parameter for defining antimicrobial efficiency [33,34], and the incorporation of polymers with high charge density as blend component will enhance the biocidal activity of the microstructured surfaces, maintaining the physicochemical properties of the resulting coating.

## 2. Experimental Section

### 2.1. Materials

Film preparation: high molecular weight polystyrene (PS, Aldrich, Schnelldorf, Germany, weight-average molecular weight, *M*_w_ = 2.50·× 10^5^ g mol^−1^) was employed as polymeric matrix and used as received. The block copolymers polystyrene-*b*-poly(4-(1-(2-(4-methylthiazol-5-yl)ethyl)-1H-1,2,3-triazol-4-yl)butyl methacrylate) (*M*_n_ = 22,000 g/mol, *M*_w_/*M*_n_ = 1.53) quaternized with either butyl iodide (PS_54_-*b*-PTTBM-B_44_) or methyl iodide (PS_54_-*b*-PTTBM-M_44_) were synthesized as previously reported [16]. Briefly, the first block of polystyrene was prepared by atom transfer radical polymerization (ATRP) and then was used as macroinitiator for the synthesis of the PTTBM block. This second block was obtained by combination of ATRP and copper-catalyzed azide-alkyne cycloaddition (CuAAC) click reaction, using the same catalyst (CuCl/PMDETA). The TTBM monomer was synthesized in situ during its ATRP polymerization by click chemistry between 2-(4-methylthiazol-5-yl)ethanol azide and hex-5-yn-1-yl methacrylate. The cationic copolymers PS_54_-*b*-PTTBM-R_44_ (R = butyl or methyl) were finally obtained by quaternization of their thiazole and triazole groups. ^1^H-NMR spectroscopy confirms that quaternization was achieved quantitatively [16]. Tetrahydrofuran (THF, Aldrich, St Quentin Fallavier, France, ACS reagent) was employed as organic solvent for the preparation of the porous films without further purification. Round glass coverslips of 12 mm diameter were obtained from Ted Pella Inc (Redding, CA, USA).

Microbiological assays: Sodium chloride (NaCl, 0.9%, BioXtra, St Quentin Fallavier, France, suitable for cell cultures) and phosphate-buffered saline (PBS, pH 7.4) were obtained from Aldrich. Sheep blood (5%) Columbia Agar plates were purchased from bioMérieux (Madrid, Spain). American Type Culture Collection (ATCC): Gram-positive *Staphylococcus aureus* (*S. aureus*, ATCC 29213) bacteria and *Candida parapsilosis* (*C. parapsilosis*, ATCC 22019) fungi were obtained from Oxoid™ (Madrid, Spain).

### 2.2. Antimicrobial Film Formation

Porous films with antimicrobial activity were fabricated by a breath figures approach. For this purpose, blends of polymers composed of commercial PS as the main component, and cationic copolymers PS_54_-*b*-PTTBM-R_44_ (PS_54_-*b*-PTTBM-B_44_ or PS_54_-*b*-PTTBM-M_44_) as minor components were prepared at different compositions: 3, 6 and 9 wt % of copolymer. The polymer mixtures were dissolved in THF at a concentration of 30 mg mL^−1^. THF was selected as organic solvent because is compatible with all the components. Then, polymer films of 1 cm diameter were obtained by drop casting of 30 µL of each solution onto glass substrate at room temperature in a closed chamber under controlled humidity. The humidity conditions of the chamber were set to 60, 70 and 90% for each solution.

### 2.3. Film Characterization

The surface structures of the films were observed by a scanning electron microscope (Philips XL30, Eindhoven, The Netherlands) with an acceleration voltage of 25 kV. The films were coated with gold-palladium (80/20) prior to imaging. The pore diameters and the quantitative order of porous patterns were analyzed by the image analysis software Image-J (NIHimage, National Institutes of Health, Bethesda, MD, USA). Water contact angle measurements of the prepared films were carried out in a KSV Theta goniometer (KSV Instruments Ltd., Helsinki, Finland) from digital images of 3.0 μL water droplets on the surface. The measurements were made in at least quintuplicate.

### 2.4. Evaluation of Antimicrobial Activity in Films

Antimicrobial activities of the prepared films were evaluated following the E2149-01 standard method from the American Society for Testing and Materials (ASTM) [35], which is a quantitative and standardized method, typically used for the analysis of material surfaces in antimicrobial materials. Firstly, the microorganisms *S. aureus* and *C. parapsilosis* were incubated on 5% sheep blood Columbia agar plates for 24 h for bacteria and 48 h for yeast at 37 °C in a Jouan IQ050 incubator (Winchester, VA, USA). Subsequently, the microorganism concentration was adjusted with saline solution to a turbidity equivalent to ca. 0.5 McFarland turbidity standard, about 10^8^ colony-forming units (CFU) mL^−1^. The optical density of the microorganism suspensions was measured in a DensiCHEKTM Plus (VITEK, bioMérieux, Madrid, Spain). Then, these suspensions were further diluted (1:200) with PBS to obtain 10^6^ CFU mL^−1^. Each film was introduced in a sterile falcon tube containing 1 mL of the tested inoculum and 9 mL of PBS to reach a working solution of ca. 10^5^ CFU mL^−1^. Control experiments were also carried out on films made exclusively with commercial PS, and also blank experiments with only the tested inoculum in the absence of films. The suspensions were shaken for 24 h at 120 rpm. After this period, 1 mL of each solution was taken and serially diluted. The dilutions, 1 mL, were placed on 5% sheep blood Columbia agar plates and incubated for 24 h for bacteria and 48 h for fungi at 37 °C. Then, the number of bacteria in each sample was determined by the plate counting method [36]. The measurements were made at least in triplicate.

## 3. Results and Discussion

**Figure 1:**
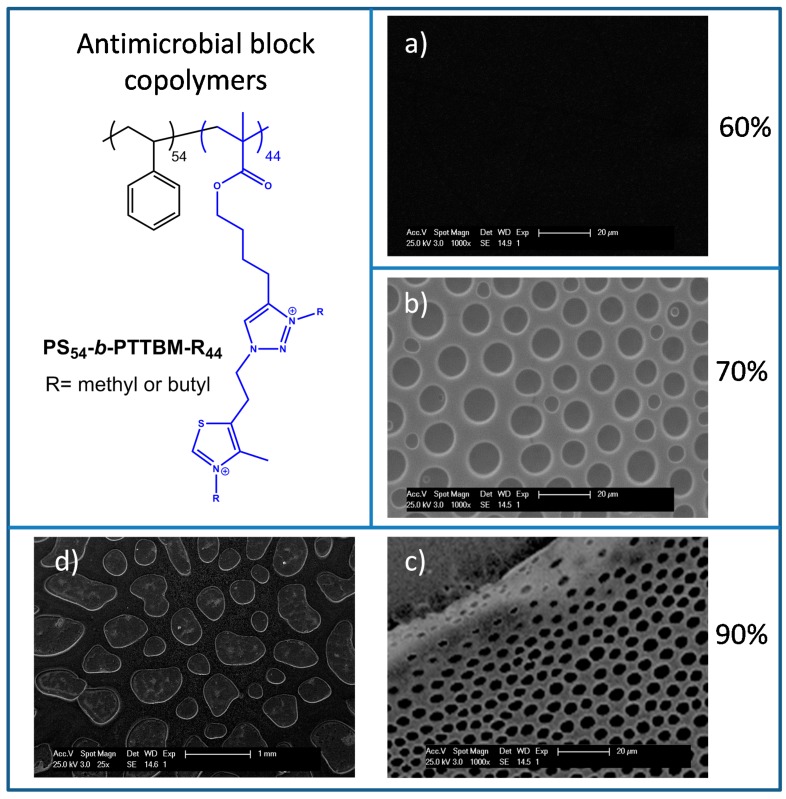
Chemical structure of the antimicrobial block copolymer quaternized with butyl (PS_54_-*b*-PTTBM-B_44_) or methyl (PS_54_-*b*-PTTBM-M_44_) iodides, and SEM images of the films of PS/PS_54_-*b*-PTTBM-B_44_ blends, 97/3 wt % obtained from THF solutions at (**a**) 60%; (**b**) 70%; and (**c**,**d**) 90% relative humidity.

Porous films were prepared by the breath figures method from blends containing antimicrobial copolymers with high charge density as a minor component or additive. By this approach, bactericidal films on contact with antimicrobial chemical functionalities at the surface and controlled microstructure can be fabricated in a very simple and effective way. In addition, structural parameters, such as pore size and pore density, can be easily controlled by selecting the experimental conditions of humidity, concentration of the solution or the type of polymer [22]. In fact, the use of polymeric structures with polar moieties, i.e., amphiphilic polymers, favors the formation of ordered porous arrays, because these structures help the stabilization of the condensed water droplets. Herein, amphiphilic copolymers based on a hydrophilic block with two quaternary ammonium moieties per monomeric unit (Figure 1) were added to commercial polystyrene as a modifier and antimicrobial component. THF solutions of these blends were cast onto glass substrates under controlled humidity. It has to be mentioned that the cationic copolymers PS_54_-*b*-PTTBM-R_44_ are not soluble in the common organic solvents typically used in the breath figures approach, such as CS_2_ and chloroform. Thus, THF was selected to be more compatible with both components of the blend, although it is known that THF is not an ideal solvent and typically leads to irregular arrays due to its miscibility with water. Nevertheless, THF only allows the solubility of low content of copolymers. For this reason, THF solution was prepared with a polymeric concentration of 30 mg/mL, with PS/PS_54_-*b*-PTTBM-R_44_ ratios of 97/3, 94/6 and 91/9 wt %.

Films were prepared from these THF solutions under different humidities: 60%, 70% and 90%. As mentioned above, the relative humidity is a fundamental parameter in the preparation of porous films by the breath figures approach and has a large impact on the morphology of the films. As shown in Figure 1, when the humidity was set at 60%, only flat surfaces were obtained; thus, in this case, higher humidity was necessary to fabricate porous films. In effect, at higher humidity values, such as 70% and 90%, porous films were found; however, films prepared at 90% show irregular patterns containing large and heterogeneous pores mixed with smaller pores, resulting from the coagulation of the rapidly condensing water droplets during the breath figure process, which leads to a dramatic increase in the droplet size (Figure 1c,d) [37]. On the other hand, when the humidity was set at 70%, more homogeneous patterns were obtained, as shown in Figure 1b. Thus, the following experiments for the fabrication of antimicrobial surfaces were carried out under this relative humidity.

**Figure 2:**
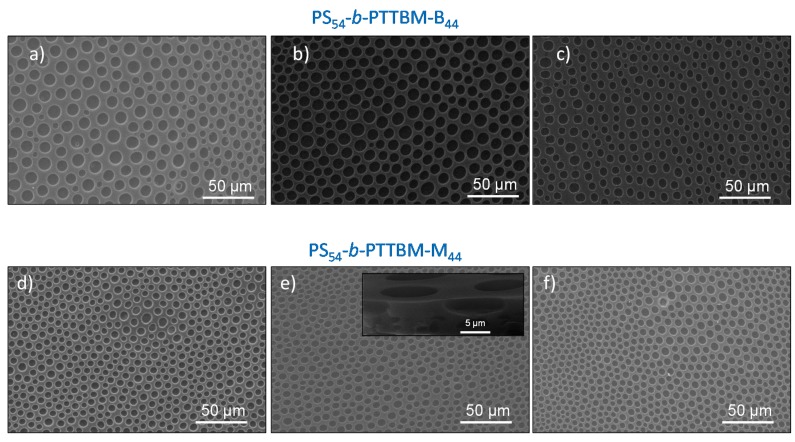
SEM micrographs of films obtained at 70% relative humidity composed of PS/PS_54_-*b*-PTTBM-B_44_ blends: (**a**) 97/3 wt %; (**b**) 94/6 wt %; (**c**) 91/9 wt %; and PS/ PS_54_-*b*-PTTBM-M_44_ blends: (**d**) 97/3 wt %; (**e**) 94/6 wt % (inset: cross-section); (**f**) 91/9 wt %.

Films were prepared at 70% relative humidity from blend solutions composed of commercial PS and a low amount of the antimicrobial copolymer quaternized with butyl or methyl iodide, PS_54_-*b*-PTTBM-B_44_ or PS_54_-*b*-PTTBM-M_44_, respectively. It is well known that the alkylating agents affect the efficacy of the antimicrobial polymers based on quaternary ammonium groups, because they modify the hydrophobic/hydrophilic balance [31,38]. Additionally, the alkylating agents would also influence the microstructure of the breath figure films. Figure 2 shows SEM images of the films containing different contents of copolymer prepared at 70% humidity, in which porous films are observed in all cases. Additionally, it is observed from the cross-section image that there is only a single layer with pores. In these SEM images, the influence of the type of copolymer and its concentration on the pore structure and morphology of the porous breath figure films can also be seen.

Previous works indicate that, in general, copolymers with large hydrophilic blocks produce poorly ordered structures because the interfacial tension tends to decrease and, consequently, the coalescence of the water droplets increases [29,37,39]. However, in this case, relatively ordered porous arrays are obtained for all the blends, using both types of antimicrobial copolymers with large cationic segments as additives. Table 1 shows the quantitative evaluation of the order obtained by using Voronoi polygon construction on low-magnification SEM images. The images were processed and analyzed by the software ImageJ to calculate the conformational entropy, which is compared with the entropy for an ideal hexagonal array (S = 0) and a randomly organized array (S = 1.71) [40]. The large entropies obtained between 1.17 and 0.86 indicate relatively poorly ordered arrays, although these values are also substantially less than S = 1.71 for random packaging. It has to be mentioned that they are typical values for breath figures made from water-miscible solvent such as THF [41,42,43]. Concerning the pore size, highly homogeneous pore diameters can clearly be seen for all the samples in SEM images. When the different films are compared, in general, higher diameters are obtained in films containing the copolymer quaternized with butyl, PS_54_-*b*-PTTBM-B_44_ (Figure 2a–c), which is more hydrophobic than PS_54_-*b*-PTTBM-M_44_ quaternized with methyl groups. Nevertheless, the differences are slight, as seen in Table 1, which summarizes the mean pore sizes of all prepared porous films, determined by measuring at least 100 pores from the SEM images. Additionally, when the concentration of both copolymers incorporated into the films is varied from 3 to 9 wt %, a slight influence is noted in the pore size, which decreases when the content of copolymer increases.

In general, we can conclude that as the hydrophilicity of the system is augmented, either by the use of more hydrophilic copolymer or by the use of more percentage of the cationic copolymer, the size of the pores decreases. It is well known in the breath figures approach that amphiphilic structures help to stabilize the water droplets condensed at the surface of the polymeric solution; thus, in polymeric blends, the content of amphiphilic copolymers significantly influences the porous structures [22]. As the content of copolymer increases in the blend, more droplets can be stabilized and, therefore, more and smaller pores can finally be formed at the surface [22,44].

The surface wettability of the films and, then, their contact with culture media mainly depends on both the chemical functionality and the roughness of the surface. The water contact angle values of obtained films were found to be ~120° for all the samples measured, independent of the copolymer content. However, as the cationic copolymer content increases in the sample, so does the hydrophilicity, and the contact angle should decrease. Therefore, the roughness of the sample, as expected, also contributes to the wettability of the films [45,46]. Table 1 summarizes the Wenzel roughness factor, r_f_, defined as the ratio between the actual and the projected areas of the surface [47]. This factor is equal to one for flat surfaces and is greater than one for rough surfaces. It is observed that the roughness of the films increases with the content of the copolymers, and in films containing the copolymer quaternized with methyl iodide. Pore diameter slightly decreases with the content of the cationic copolymer, but at the same time, pore density also increases, which contributes to the augmentation of the roughness. These contrary contributions to wettability, chemical functionality and roughness could be the reason for the similar contact angles values found in the films. Therefore, in principle, microbial contact with the surface would be rather similar for all the samples.

The antimicrobial activity of the prepared breath figure films was evaluated against *S. aureus* Gram-positive bacteria and the fungi *C. parapsilosis* as model microbes, since they are common pathogens responsible of many nosocomial infections. The shake flask method [36] was employed to quantify the antimicrobial activity of the films under dynamic contact conditions. Table 2 summarizes the cell killing percentage in microbial medium in contact with the films for 24 h, and then the growth in agar plates for 24 h and 48 h for bacteria and fungi, respectively. The cell killing percentages were expressed with respect to control experiments in which the microbial reduction was null (experiments performed with films prepared from commercial PS, 0 wt % of copolymers, and without any films).

It can be seen that all films exhibit high killing efficiency against *S. aureus* bacteria, with a reduction of more than 99.99% in the culture medium. On the other hand, moderate activity was found against *C. parapsilosis* fungi, with reduction of up to 90% for contents of copolymer higher than 6%. It is worth mentioning that these films present relatively high antimicrobial activity even with very low content of cationic copolymer; films containing only 6 wt % copolymers can reduce 99.99% of *S. aureus* and 90% of *C. parapsilosis* exposure to the films. Thus, these results reveal that the preparation method provides films with enough accessible active groups at the surfaces to kill the microorganisms by surface contact, even when low amounts of copolymer are incorporated in the film. Remarkably, these breath figure films provide better efficiencies than flat films prepared directly from the copolymer solution; that is, 100 wt % of PS_54_-*b*-PTTBM-B_44_, PS_54_-*b*-PTTBM-M_44_ [16]. These findings demonstrate the importance of the surface roughness on the antimicrobial activity of contact-active films, which allows the use of very low amounts of antimicrobial component in the coating while maintaining excellent biocidal activity.

## 4. Conclusions

In summary, efficient antimicrobial porous coatings were fabricated by the breath figures approach from blends containing very low contents of antimicrobial polymers. Highly active amphiphilic copolymers with a large cationic block bearing a flexible side chain with 1,3-thiazolium and 1,2,3-triazolium groups were used as antimicrobial polymers with high charge density. Due to the high biocidal effectiveness of the copolymers and the controlled roughness of the porous surfaces, the resulting films exhibit high killing efficiency against the studied microorganisms. Thus, we can conclude that this breath figures approach, using only a low content of cationic polymers, allows the formation of surfaces with accessible polycationic chains for killing the microorganisms *S. aureus* and *C. parapsilosis* by surface contact.

## Figures and Tables

**Table 1:** Average pore diameter ± SD (standard deviation), conformational entropy (S), and roughness factor (r_f_) of porous films containing variable content of the cationic copolymers PS_54_-*b*-PTTBM-B_44_ and PS_54_-*b*-PTTBM-M_44_.

Cationic Copolymer	Concentration (wt. %)	S	Pore Size (µm)	r_f_
PS_54_-*b*-PTTBM-B_44_	3	1.16	11 ± 1	1.33
	6	0.91	10 ± 1	1.42
	9	1.17	7 ± 1	1.41
PS_54_-*b*-PTTBM-M_44_	3	0.86	7 ± 1	1.47
	6	0.98	6 ± 2	1.48
	9	0.94	5 ± 1	1.48

**Table 2:** Cell killing percentage of the breath figure films for *S. aureus* and *C. parapsilosis* microorganisms.

Cationic Copolymer	Concentration (wt %)	Cell Killing (%)	
		*S. aureus*	*C. parapsilosis*
PS_54_-*b*-PTTBM-B_44_	3	99.99	50
	6	99.99	90
	9	99.99	90
PS_54_-*b*-PTTBM-M_44_	3	99.99	90
	6	99.99	90
	9	99.99	90
